# Combination of Sensory, Chromatographic, and Chemometrics Analysis of Volatile Organic Compounds for the Discrimination of Authentic and Unauthentic Harumanis Mangoes

**DOI:** 10.3390/molecules23092365

**Published:** 2018-09-16

**Authors:** Siti Raihan Zakaria, Norashikin Saim, Rozita Osman, Zaibunnisa Abdul Haiyee, Hafizan Juahir

**Affiliations:** 1 Faculty of Applied Sciences, Universiti Teknologi MARA Pahang, Jengka 26400, Pahang, Malaysia; sitiraihan@pahang.uitm.edu.my; 2 Faculty of Applied Sciences, Universiti Teknologi MARA (UiTM), Shah Alam 40450, Selangor, Malaysia; rozit471@salam.uitm.edu.my (R.O.); zaibunnisahaiyee75@gmail.com (Z.A.H.); 3 East Coast Environmental Research Institute, Universiti Sultan Zainal Abidin, Kuala Terengganu 21300, Malaysia; hafizanjuahir@unisza.edu.my

**Keywords:** Harumanis, *Mangifera indica* L., VOCs, sensory analysis, chromatographic, chemometrics, authentication

## Abstract

This study analyzed the volatile organic compounds (VOCs) of three mango varieties (Harumanis, Tong Dam and Susu) for the discrimination of authentic Harumanis from other mangoes. The VOCs of these mangoes were extracted and analysed nondestructively using Head Space-Solid Phase Micro Extraction (HS-SPME) coupled to Gas Chromatography-Mass Spectrometry (GC-MS). Prior to the analytical method, two simple sensory analyses were carried out to assess the ability of the consumers to differentiate between the Harumanis and Tong Dam mangoes as well as their preferences towards these mangoes. On the other hand, chemometrics techniques, such as principal components analysis (PCA), hierarchical clustering analysis (HCA), and discriminant analysis (DA), were used to visualise grouping tendencies of the volatile compounds detected. These techniques were successful in identifying the grouping tendencies of the mango samples according to the presence of their respective volatile compounds, thus enabling the identification of the groups of substances responsible for the discrimination between the authentic and unauthentic Harumanis mangoes. In addition, three ocimene compounds, namely *beta*-ocimene, *trans beta*-ocimene, and *allo*-ocimene, can be considered as chemical markers of the Harumanis mango, as these compounds exist in all Harumanis mango, regardless the different sources of the mangoes obtained.

## 1. Introduction

The mango (*Mangifera indica* L.) is one of the most popular fruit worldwide and is grown in more than 100 countries, most of which are in Asia [1]. It is known as ‘the King of Fruit’ due to its attractive colour, delicious taste, and exotic flavour. Apart from that, the mango is also rich in nutrients and minerals such as magnesium, potassium, sulphur, phosphorus, and vitamins A, C, and D [2]. The characteristic taste of the mango is mostly attributable to the types and amounts of sugars and organic acids, while volatile compounds distinguish its flavour.

**Figure 1:**
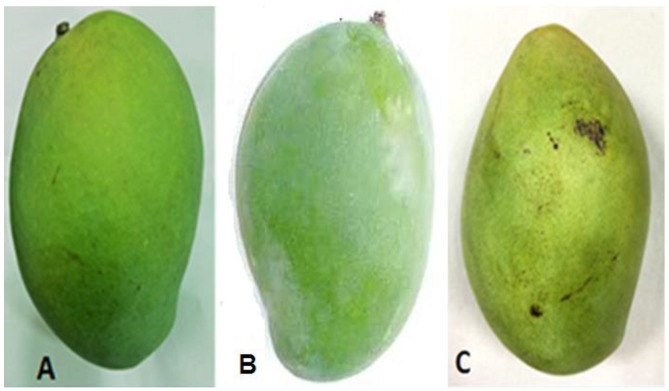
Harumanis (**A**), Tong Dam (**B**) and Susu (**C**) mangoes.

In Malaysia, the exotic aroma of the Harumanis mango (MA128) has become the trademark of the fruit and is prized by many consumers [3]. The Harumanis is often highly valued not only because of its delicious taste, but also because it is considered to be rare and limited and can only be found in Perlis, Malaysia, and Surabaya (Indonesia). Due to its high price and popularity, the Harumanis has become the target of economic adulteration involving substitution and misinterpretation. The media has constantly reported the unethical activities of sellers who substitute the Perlis premium Harumanis mango with cheaper mangoes (Tong Dam and Susu) to gain fraudulent profits. According to the Perlis Department of Agriculture, Tong Dam and Susu mangoes are often used to deceive consumers because these mangoes have physical characteristics that are almost identical to the Harumanis mango, as shown in Figure 1, and therefore are called unauthentic Harumanis. In addition, the prices of these unauthentic Harumanis mangoes are five times cheaper than the Harumanis mango itself and this fact entices errant traders to replace the Harumanis with the Tong Dam to gain bigger profits. Thus, a comprehensive tool that can be used to differentiate between the authentic and unauthentic Harumanis mangoes is crucial.

Extensive chemometric tools comprising both supervised and unsupervised techniques have been utilized for food discrimination, authentication, and origin traceability [4,5,6]. The supervised multivariate classification technique refers to a mathematical model that has been developed by using a training data set or calibration data that is able to classify and differentiate future samples, whereas the supervised technique utilises the complex data’s pattern recognition. Principal component analyses (PCA), neural networks, discriminant analyses (DA), hierarchical cluster analysis (HCA), and partial least square regression (PLSR) are among the common chemometric methods that are used together with the flavour fingerprint data to visualize the fingerprint’s information [7,8,9]. These methods can be considered as the convenient tools to visualize even the minor differences among similar chromatograms and provide a reasonable definition [10]. For instance, the aroma volatile profiles in different pomegranate cultivars have been studied by Beaulieu and his co-researchers by using Gas Chromatography-Mass Spectrometry (GC-MS) for the analysis and PCA as the tool to differentiate and classify the pomegranate samples according to their cultivars [11]. PCA had successfully differentiated the pomegranate according to cultivar, as there were variations in the volatile profile of the pomegranate in different cultivars, and the researchers found that aldehyde and terpene concentrations could be used to characterise the pomegranate cultivars.

The purpose of this study was to discriminate the Harumanis mango from the other mangoes by extracting the VOCs in the mangoes using HS-SPME followed by the separation and identification of the compounds using GC-MS. In order to successfully differentiate and discriminate between the authentic and unauthentic Harumanis mangoes, the multivariate chemometric techniques (PCA, HCA, and DA) were performed. In addition, two simple sensory analyses, namely the duo trio test and the acceptance test, were also conducted to evaluate the panels view on the mangoes tested. These tests were carried out prior to the volatile compounds analysis to observe the significant difference between the Tong Dam and Harumanis mangoes and to evaluate the preferences of the panelists.

## 2. Results and Discussion

### 2.1. Sensory Analysis

Sensory analyses were carried out to evaluate the ability of the respondents to differentiate between the authentic and unauthentic Harumanis mangoes. In these analyses, only the Tong Dam mango was used as a representative of the unauthentic Harumanis mangoes, because according to Perlis Department of Agriculture, the Tong Dam mango is the unauthentic Harumanis that most resembles the authentic Harumanis mango. In addition, at the time the sensory analyses were carried out, Susu mangoes were not available in the market yet; thus, the sensory analyses were only done between the Tong Dam and Harumanis mangoes. The first sensory analysis done was a simple difference test called ‘Duo-Trio Test’: this was performed on 30 respondents whereby two mangoes (Harumanis and Tong Dam) were given to be evaluated along with a reference sample (Tong Dam). Both Harumanis and Tong Dam mangoes were labeled with different codings, and the respondents were asked to choose which sample was identical to the reference sample. The result obtained in Table 1 showed that 17 out of 30 respondents managed to choose the correct sample that matched the reference sample (which was the Tong Dam samples), whereas 43% of the respondents got the wrong answer by choosing the Harumanis mango. This test gave an insignificant value, suggesting the Harumanis and Tong Dam mangoes were difficult to be differentiated physically by a lay person.

In addition, the acceptance tests that evaluated the preferences of the respondents towards the authentic and unauthentic Harumanis were also carried out using a 9-point hedonic sensory evaluation. Two tests were done using whole and slices of Harumanis and Tong Dam mangoes. In this test, the respondents were given a 9-point hedonic sensory evaluation that comprised a few questions to assess their preferences towards these mangoes. Five parameters were evaluated using whole mango samples which were colour, aroma, physical appearance, texture and overall acceptability. On the other hand, taste, colour, aroma, texture and overall acceptability were the parameters evaluating the respondents’ preferences of the slices of mangoes samples.

**Figure 2:**
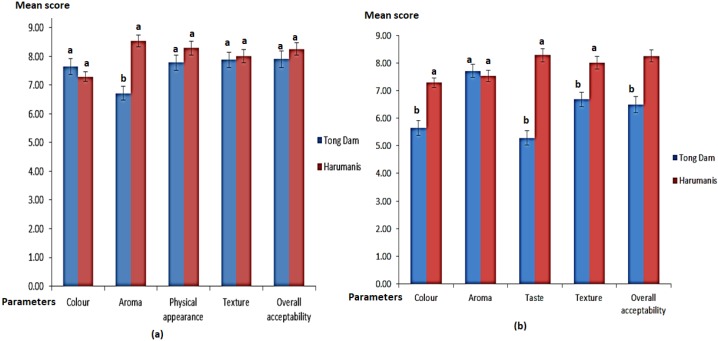
Acceptance test of the parameters for (**a**) whole Harumanis and Tong Dam samples and (**b**) slices of Harumanis and Tong Dam samples, evaluated using a 9-point hedonic sensory evaluation. Different letters indicate significant differences of mean concentrations (*p* ≤ 0.05) between the two mango cultivars.

As shown in Figure 2a, only aroma recorded a significant difference between the authentic Harumanis and Tong Dam samples, while the rest of the parameters showed an insignificant difference. The significant differences of the two samples were denoted by the difference of the alphabets used [12]. This result once again suggested that the Tong Dam and Harumanis mangoes were almost identical and can only be differentiated using an aroma parameter. Apparently, this was in line with the suggestion from the Department of Agriculture in Perlis, which asked consumers to buy only ripe Harumanis, given that the aroma is the most significant parameter to differentiate between the authentic and unauthentic Harumanis mangoes. On the other hand, when slices of both mangoes were sampled in the preference test, the outcome had different findings, as shown in Figure 2b. The four parameters of colour, taste, texture, and the overall acceptability of the product were significantly different, suggesting that the slices of Harumanis mangoes were tastier to the consumers than the Tong Dam mangoes. The three sensory analysis results showed quite clearly that the tendency of the consumers to be misled by irresponsible sellers is high, because the unauthentic and authentic Harumanis mangoes are almost identical, with only the aroma as a significant parameter that can differentiate the two varieties. Thus, a more efficient technique is required.

### 2.2. Gas Chromatography Fingerprint

**Figure 3:**
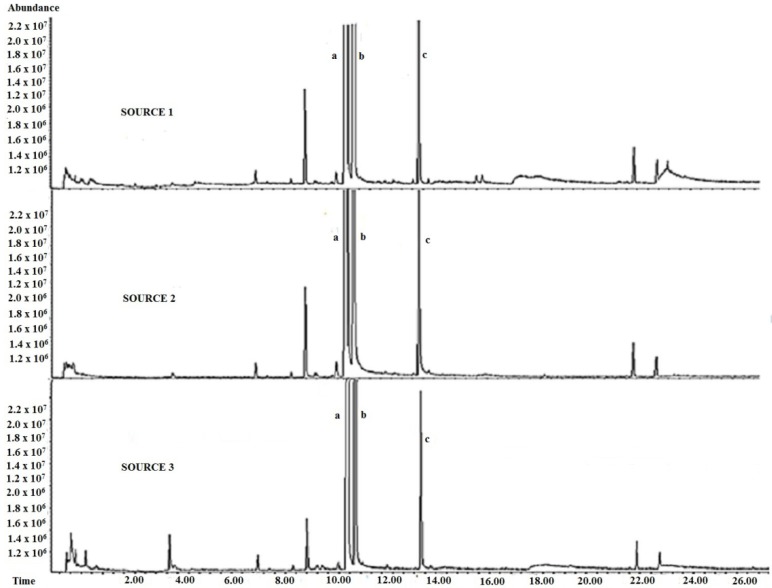
Chromatograms of the real Harumanis mango in three locations with three marker compounds. (a) *beta*-ocimene, (b) *trans-beta*-ocimene and (c) *allo*-ocimene.

The separation and identification of the mangoes’ VOCs were performed using GC-MS. A total of 123 VOCs was identified based on the comparison of mass spectra of unknown with the NIST library. Figure 3 shows the chromatograms of Harumanis mangoes from three sources in Perlis. All chromatograms showed similar pattern with three significant chemical markers that can only be found in Harumanis mangoes which were *beta*-ocimene, *trans-beta*-ocimene, and *allo*-ocimene at these retention times 11.144 min, 11.418 min, and 13.874 min, respectively.

**Figure 4:**
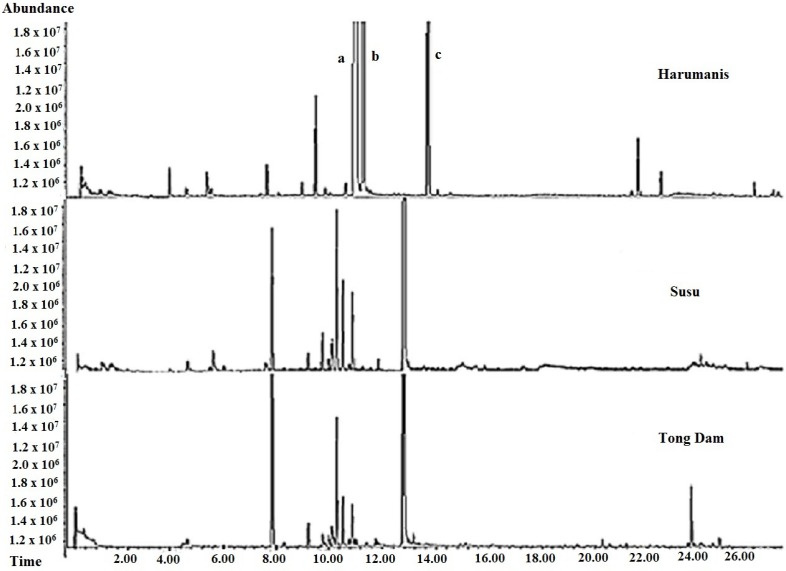
Chromatograms of Harumanis, Susu, and Tong Dam mangoes with three chemical compounds appearing in Harumanis: (a) *beta*-ocimene, (b) *trans-beta* ocimene and (c) *allo*-ocimene.

Two of these chemical markers, *beta*-ocimene and *trans-beta*-ocimene, had been reported in a previous study [13]. Meanwhile, Figure 4 showed the comparisons of the chromatograms for the three varieties of mangoes. Surprisingly, the chromatograms for both of unauthentic Harumanis mangoes, Tong Dam and Susu, showed similar pattern, whereas the chromatogram of the authentic Harumanis showed a significantly different pattern. In addition, the three chemical markers of Harumanis were not found in either Tong Dam or Susu mangoes.

### 2.3. Cluster Analysis (CA)

Cluster Analysis (CA) is often used to classify large samples into clusters with high similarities within the class and high dissimilarity between different classes. In this study, a CA was applied to 26 selected compounds with Relative Percent Area (RPA) quality above 80% for the pattern recognition of 10 batches of the authentic Harumanis mangoes and 13 batches of the unauthentic Harumanis, comprising 7 batches of Tong Dam and 6 batches of Susu mangoes, respectively, using between-groups linkages as an amalgamation rule (similarities) and Euclidean distance metric (dissimilarities). These methods had been successfully used in classifying and clustering samples such as tongkat ali roots [14], the Alphonso mango [15], and the Nam Dok Mai mango [16].

**Figure 5:**
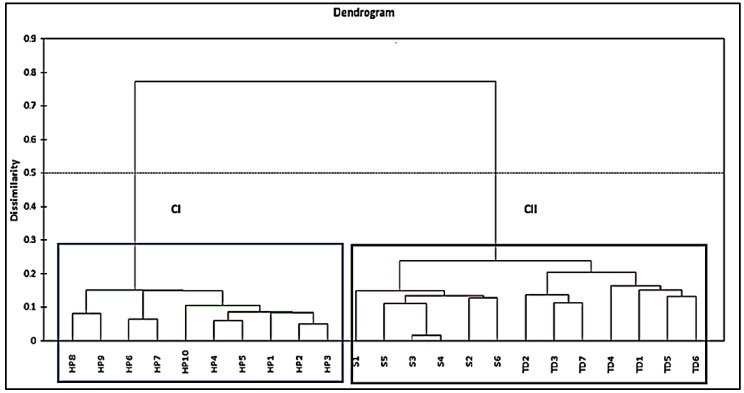
Dendrogram of the authentic (CI) and unauthentic (CII) Harumanis mango.

The dendrogram (Figure 5) visibly showed that all samples were divided into two major clusters, suggesting that the samples in different clusters have significant difference [17]. As expected, all the authentic Harumanis mangoes were grouped in Cluster I suggesting that all the samples contained high similarity of the VOCs. In Cluster I, there were three different mini clusters which corresponded to different sources of Harumanis. Saim et al. [18] stated that the mini clusters in the main cluster showed only slight differences but higher similarities, as compared with main different clusters. In addition, the dendogram showed that the unauthentic Harumanis mangoes were in a different cluster than the real Harumanis, confirming that both authentic and unauthentic Harumanis had high chemical dissimilarity. On the other hand, below 0.5 dissimilarity, both Tong Dam and Susu mangoes were grouped in the same cluster (Cluster II).

### 2.4. Discriminant Analysis (DA)

Discriminant analysis was performed using a set of observations for which the classes are known. In this study, the predefined classes were 2 (authentic and unauthentic Harumanis mango) and were obtained from the CA. This set of observations is sometimes referred to as the training set. A Linear discriminant analysis (LDA) was used to set a linear function of variables. A confusion matrix was used as the validation method. As in a CA, 26 selected compounds were used to determine whether groups differ with regards to the mean of a variable, and to use that variable to predict group membership. A DA can also determine the variables that discriminate samples between clusters in the CA. The results of DA, as shown in Table 2, showed the classification matrix for discriminant analysis of VOCs in Harumanis, Tong Dam, and Susu mangoes, using a standard DA mode that gave a 100% correct classification of the mangoes according to the varieties. The DA showed that each group differed from the others in terms of different compositions, as no confusion matrix occurs on the data set [19].

### 2.5. Principal Component Analysis (PCA)

PCA is a powerful tool to reduce the dimensions of multivariate data set. In this study, PCA was applied to determine in what respect one sample is different from another and which variables contribute most to this difference. Similar to the DA, 26 compounds were used as the variables.

**Figure 6:**
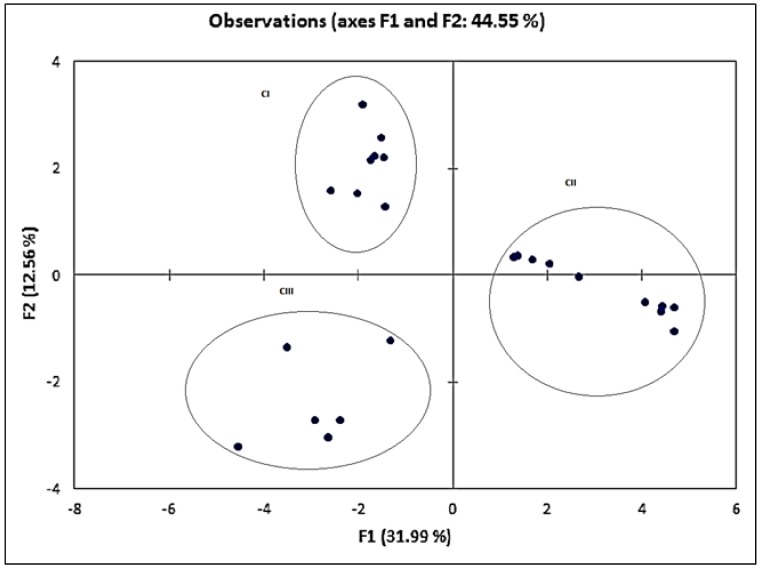
PCA plots of the three mangoes varieties; Susu (CI), Harumanis (CII), and Tong Dam (CIII).

Figure 6 shows that all the three mango varieties were distinctively separated. The first two factors at 31.99% and 12.56% of variation were selected to provide the highest variation of data for convenient visualization and differentiation. The result suggested that each mango variety had their own unique chemical compositions [20]. The results presented by the PCA also indicated that there was a greater dispersion among the samples of Harumanis mango, which was consistent with the dendrogram in CA and can be explained by the higher quantity of compounds detected in smaller concentrations. The samples of the unauthentic Harumanis presented a higher clustering tendency and were located closely and in the same vertical axis. In addition, the agreement between CA and DA is consistent with this idea. 

Table 3 tabulated PCA loadings for mango samples having a total variance of 77.148% where seven factors were extracted with contributions of 31.994%, 12.558%, 8.733%, 7.151%, 6.479%, 5.1935%, and 5.0408%, respectively. The strong loadings of all the ocimene compounds (*beta*-ocimene, *trans-beta*-ocimen, and *allo*-ocimene) and factors 1 and 3 confirmed that these compounds can be the chemical markers for Harumanis authentication. In addition, *beta*-myrcene and heicosene also recorded strong loadings in factor 1.

## 3. Materials and Methods

### 3.1. Analysis of Volatiles

The Harumanis mangoes were obtained from local orchards in Perlis, Malaysia with the assistance of Agriculture Officer from Perlis Department of Agriculture, Malaysia. All fruits were in full maturity. Harumanis mangoes were considered ripe after 56 days of blooming. Ripe Tong Dam and Susu mangoes were also obtained from local markets in Perlis, Malaysia. All mango samples collected were about similar sizes and weights (350–450 g). For the volatile analysis of the whole mangoes, the mangoes were washed thoroughly with tap and distilled water, dried and were run immediately (within three days of sampling) to avoid the further ripening of the mangoes. The mango was placed in specially made glass vials covered with septa and left for 2 h to allow the VOCs to vaporise naturally from the mangoes. After 2 h, the vial containing the mango was placed in the water bath at 40 °C and the VOCs were extracted using polydimethylsiloxane/divinyl fiber (PDMS/DVB (Polydimethylsiloxane/Divinylbenzene), Stable Flex^®^, Supelco, Bellefonte, PA, USA) for 20 min. All analyses were carried out in duplicates.

The volatile compounds were analyzed using a GC-MS system (Agilent Technologies 5973 Inert Mass Selective Detector and Agilent 7683 Series, Palo Alto, CA, USA) under the following operating conditions: HP-5MS capillary column (Agilent 19091S-433, 30 m × 0.25 mm × 0.25 µm) was used. GC-MS detection was done using an electronionization system with ionization energy of 70 eV in the 50–550 a.m.u. mass range. Column temperature was increased from 40 °C to 280 °C with temperature program: 40 °C–100 °C (2 °C·min^−1^)–160 °C (6 °C·min^−1^) and 280 °C (15 °C·min^−1^); injector temperature and mode: 280 °C, split ratio: 1:100; ion source temperature: 230 °C and interface temperature: 280 °C. Helium was the carrier gas, at a flow rate of 1.2 mL·min^−1^. The VOCs in Harumanis mangoes were identified by comparing their spectra with those available in the NIST digital library.

### 3.2. Sensory Analysis

Two simple sensory analyses, namely the duo trio test and preference test, were carried out prior to the analytical experiments to assess the ability of the consumers to differentiate between Harumanis and Tong Dam mangoes as well as their preferences towards these mangoes. The preference test used the 9-point hedonic scale. The parameters assessed were colour, aroma, physical appearance, texture, and overall acceptability of authentic Harumanis and Tong Dam (unauthentic Harumanis) mangoes. Consumers evaluated samples based on overall liking using a 9-point hedonic scale (1 = dislike extremely; 2 = dislike very much; 3 = dislike moderately; 4 = dislike slightly; 5 = neither like nor dislike; 6 = like slightly; 7 = like moderately; 8 = like very much; 9 = like extremely) [21]. Thirty untrained adult consumers were selected to perform the test and the experiments were carried out in a closed cabin with white illumination. The samples were labeled with three random digits on a white surface. These samples had a monadic form and followed a balanced order of presentation [22].

### 3.3. Data Analysis

The chemometric techniques of cluster analysis (CA), principal component analysis (PCA), and discriminant analysis (DA) were performed using XLSTAT Software (XLSTAT, 2015, Addinsoft, New York, NY, USA). For the analysis of sensory analysis data, significant differences of means were determined by analysis of variance (ANOVA) and the data sets with normal distribution and homogeneous variance were compared using Tukey’s multiple range test (*p* ≤ 0.05).

## 4. Conclusions

The HS-SPME coupled with GC-MS was successful in extracting, separating, and identifying 123 VOCs from three mango varieties, Harumanis, Tong Dam, and Susu mangoes. A total of 26 compounds was selected (RPA quality greater than 80) and subjected to chemometric analysis. The duo trio sensory analysis test carried out on 30 respondents showed that only 57% of the respondents managed to differentiate between the whole Harumanis and Tong Dam mangoes and the significant parameter recorded was only the aroma. The chemical fingerprinting analysis showed similar patterns in the Harumanis mango from different sources with three significant compounds discovered which were *beta*-ocimene, *trans-beta*-ocimene, and *allo*-ocimene. These three ocimene compounds can be considered as the chemical markers for Harumanis mango as these compounds exist in all Harumanis mango, regardless of the different sources of the mangoes obtained with the same retention times; these were 11.144 min, 11.418 min, and 13.874 min, respectively. In addition, all these ocimene compounds did not appear in the unauthentic Harumanis volatile profile. Nevertherless, these ocimene compounds also appeared to have strong loadings (more than 0.75) along with *beta*-myrcene and heicosene. The multivariate analysis namely CA, DA, and PCA were successful in identifying the grouping tendencies of the mango samples according to the presence of their respective volatile compounds, thus enabling the identification of the groups of substances responsible for the discrimination between the authentic and unauthentic Harumanis mangoes.

## Figures and Tables

**Table 1:** Number of agree judgment of respondents (n = 30) on a Duo Trio test.

Reference	Number of Agree Judgments	
Tong Dam	Tong Dam	Harumanis
30	17	13

**Table 2:** Classification matrix of VOCs in different variety of mangoes.

Source	% Correct	Source Assigned by DA		
		Harumanis	Susu	Tong Dam
Standard DA mode				
Harumanis	100	10	0	0
Susu	100	0	6	0
Tong Dam	100	0	0	7
Total	100	10	6	7

**Table 3:** Loadings of volatile organic compounds (VOCs) for Harumanis mango samples from point sources.

Parameters	F1	F1	F1	F1	F1	F1	F1
ethyl acetate	*0.5989*	−0.0668	0.1046	−0.1006	0.3285	0.2685	0.0473
cyclotrisiloxane	−0.2479	*0.5122*	0.0398	−0.1300	0.0020	−0.0686	0.0354
1*R*-*alpha*-pinene	*0.5844*	−0.0557	−0.4895	0.1708	−0.5931	−0.0475	−0.0058
2,6,6-Trimethyl	−0.4089	0.6959	0.1444	0.0552	0.0072	−0.2591	−0.2182
camphene	*−0.5717*	−0.6812	−0.0450	−0.1776	0.0228	−0.2657	0.2247
*beta*-pinene	*−0.5727*	−0.5703	−0.0208	−0.1859	0.0216	−0.3885	−0.0783
*beta*-myrcene	**0.7863**	−0.1486	0.1526	0.0816	−0.175	−0.0822	−0.0206
hexanoic acid	0.4131	−0.0892	−0.2776	0.2624	*−0.5664*	−0.2539	−0.047
(+)-3-carene	*−0.5509*	0.4894	0.1065	−0.0780	0.0180	−0.3210	−0.4591
3-heptene	−0.2235	*0.5403*	0.0822	−0.0053	0.0053	−0.1772	0.4339
(+)-4-carene	*−0.5356*	−0.6359	0.0472	0.1751	−0.0037	−0.1551	0.2726
1,3-cyclohexadiene	*−0.6668*	0.0028	0.2105	0.5431	−0.0393	0.0828	−0.0138
*o*-cymene	*−0.6223*	−0.0689	0.2047	0.5145	−0.0273	−0.1676	0.3267
*trans-beta*-ocimene	0.4162	−0.1325	**0.8085**	−0.2094	−0.2824	0.0615	0.0085
*beta*-ocimene	0.4108	−0.133	**0.8144**	−0.2078	−0.2764	0.058	0.0078
*gamma*-terpinene	−0.445	−0.1911	0.1321	0.4781	−0.0441	*0.5204*	−0.3439
undecane	−0.4981	*−0.5488*	−0.0366	−0.0694	0.0025	0.3551	−0.1115
*allo*-ocimene	**0.8647**	−0.1314	−0.2506	0.2139	−0.1572	−0.1157	−0.0287
octanoic acid	*0.5698*	−0.1241	−0.0111	0.2726	0.54	−0.2734	−0.062
ethyl (*E*)-2-octenoate	*0.5551*	−0.0501	−0.3193	0.0568	0.1252	0.2036	0.0299
decanoic acid	*0.6249*	−0.1302	−0.0043	0.215	0.6032	−0.0902	−0.0292
*gamma*-murrolene	*−0.3906*	0.1662	0.0649	0.048	0.0043	0.105	0.5499
germacrene D	*0.7344*	−0.1102	0.2001	0.2503	0.1101	−0.2713	−0.0247
heneicosane	**0.8052**	−0.1997	0.4348	0.1317	0.0326	−0.2029	−0.0434
Eicosane	*−0.609*	−0.1034	0.2084	0.5369	−0.0353	−0.1027	−0.2779
eigenvalue	8.3184	3.265	2.2706	1.8592	1.6844	1.3503	1.3106
variability (%)	31.9939	12.5577	8.733	7.1509	6.4786	5.1935	5.0408
cumulative (%)	31.9939	44.5515	53.2845	60.4354	66.9141	72.1076	77.1484

Note: Strong loadings (>0.75) are shown in bold and moderate loading (0.5–0.75) in italic.
